# Narrowing the niche of shark fin harvests in the global ocean

**DOI:** 10.1098/rsbl.2021.0206

**Published:** 2021-07-14

**Authors:** Kyle S. Van Houtan, Gabriel Reygondeau, Tyler O. Gagné, Kisei R. Tanaka, Salvador J. Jorgensen, Stephen R. Palumbi

**Affiliations:** ^1^ Monterey Bay Aquarium, Monterey, CA 93940, USA; ^2^ Nicholas School of the Environment, Durham, NC 27708, USA; ^3^ Changing Ocean Research Unit, Institute for the Oceans and Fisheries, University of British Columbia, Vancouver, British Columbia, Canada, V6T 1Z4; ^4^ Institute of Marine Science, University of California, Santa Cruz, CA 95060, USA; ^5^ Department of Biology, Hopkins Marine Station, Stanford University, Pacific Grove, CA 93950, USA

**Keywords:** species distributions, wildlife trade, geographic range, shark conservation

In their Comment [1], Raoult *et al.* challenge our use [2] of species distribution models (SDMs) to inform on the geographical origins of shark fins sold in global markets. This is despite our primary result that shark conservation should prioritize areas within the Exclusive Economic Zones (EEZs); a conclusion supported by independent biogeographic analyses [3,4], reconstructed global fishing effort and catch [5,6], and syntheses of biotelemetry data [7]. Our analysis also responds to calls for new approaches [8,9], as official fin trade statistics have established flaws [10] that miss what remains illegal or unreported. The main criticism of Raoult *et al.* is that our SDMs make unrealistic assumptions about fishing and that our SDMs disagree with published expert range maps (‘ERMs’, e.g. [11,12]). For four species of sharks, Raoult *et al.* compare our SDMs to ERMs, claiming the ERMs represent ‘established geographical distributions’. We address these points below.

To begin, it is important to note some limitations of ERMs and their application. ERMs are often generated without transparent and standardized data pipelines, may lack rigorous quality control frameworks, and can be slow to incorporate new information. The ERM sources the authors cite [11,12] were last updated in 2013, do not offer digital shapefiles of the data, and many of the maps have stated ambiguities and remain unchanged from decades previous. Beyond this, ERMs are inherently binary in nature and present no information on the population density or geographical affinities within a species range, further suggesting a species has zero occurrence outside [13]. Such claims are consistently challenged by electronic tagging and fisheries data. One example is with Atlantic bluefin tuna (*Thunnus thynnus*), where the United Nations Food and Agriculture Organization's ERM does not include regions with SDM predictions or public fishery records [14,15].

By comparison, our SDMs are generated from 805 235 observations obtained from living public databases—the Global Biodiversity Information Facility, FishBase, and the Ocean Biodiversity Information System [16–18]—which have extensively curated and contemporary data. Following best practices for SDM [19,20], our analysis [2] detailed a rigorous process of data screening and model validation using 15-fold 70/30 spatial cross validations, where the average area under the curve (AUC) criteria from all folds must exceed 0.80. This means that the data for model testing and calibration are independent, and that SDMs must accurately discriminate 80% of the training data. Truthfully, SDMs can be challenged by the quality of observation data, projecting fundamental niches that are not realized, and spatial non-stationarity. Therefore, we developed an ensemble modelling framework to address potential bias and spurious observations. Our resulting SDMs are built from publicly available observation data, and do not generate habitat in regions without verified observations.

We employed SDMs in this setting because they have found important applications in fisheries science and management. The United States (US) National Marine Fisheries Service, for example, couples SDMs of protected species with real-time environmental data to inform fishery operators where bycatch risk is highest [21]. Another study generated SDMs from pelagic longline vessels (pretending they were ‘apex marine predators') and finding the vessels and their target prey shared a similar environmental niche [22]. Following these important examples, our analysis assumes fishing effort has a non-uniform environmental niche that parallels target species and their habitat preferences. Advances in the availability of occurrence data and modelling approaches have made integrating SDMs into ocean management a powerful new tool, as niche models have been validated to inform on the spatial overlap of fishery operators and key species.

Figure 1*a,b* compare the observation records used to feed our SDMs to published ERMs from the International Union for Conservation of Nature (‘IUCN’, [23]) for *Carcharhinus amblyrhynchos*, *Isurus oxyrinchus, I. paucus*, and *Sphyrna tudes*. (We replaced *Sphyrna mokarran* with *I. paucus* as *S. mokarran* had multiple ERMs.) Figure 1*a* counts the percentages of observations that are within and outside the ERM. For these species, the number of observed occurrences that falls outside the ERM ranges from 9.3% to 95.9%. For coastally restricted species such as *C. amblyrhynchos* and *S. tudes,* public databases provide validated empirical observations in novel marine regions. Subjectively censoring such data sources falsely assumes that species ranges are either fixed or already perfectly understood [24]. For widespread pelagic species such as *I. oxyrinchus* and *I. paucus*, most extra-range observations are adjacent the ERM, perhaps reflecting more recent poleward expansions from ocean warming [24,25]. Figure 1*c* crops our SDM output for *I. paucus* with its corresponding ERM, emphasizing the structure of habitat preferences the SDM provides that is missing from the comparatively flat ERM.

**Figure 1.**
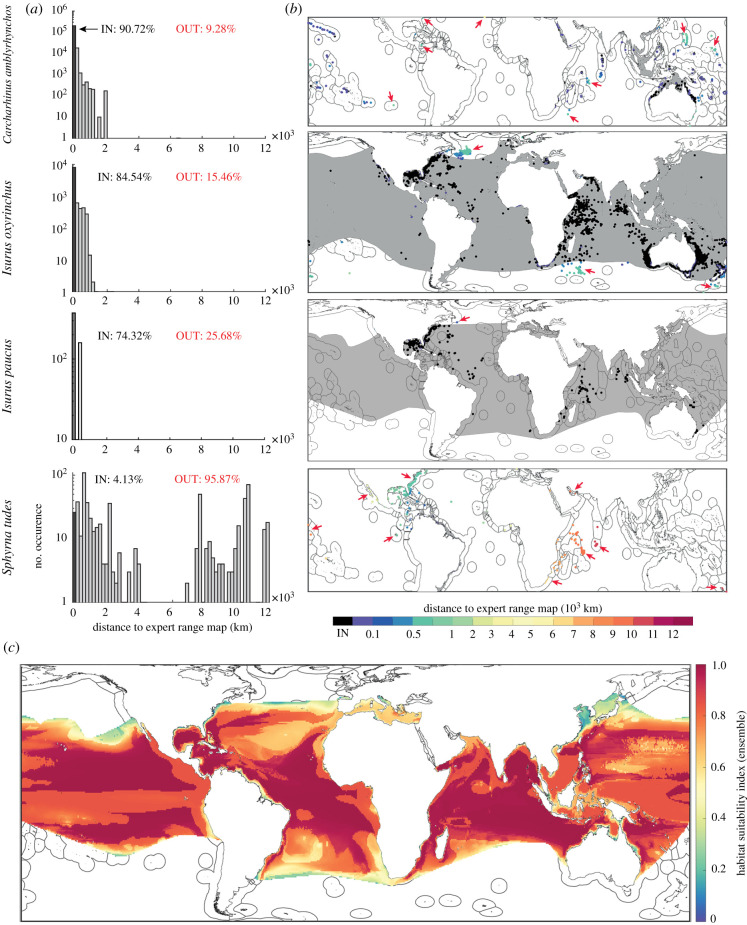
Comparing SDMs and expert range maps (ERMs). (*a*) ERMs perform variously, but never contain the full array of validated observations from third party databases of animal observations [16–18]. (*b*) Occurrences are colour-coded by their distance to the ERM (black is within the ERM). *Carcharhinus amblyrhynchos* and *S. tudes* both have validated empirical observations in novel ocean regions, where *I. oxyrinchus* and *I. paucus* have confirmed occurrences immediately adjacent to the ERM polygons. Red arrows denote clusters or notable observations outside the ERM polygon. (*c*) The derived SDM for *I. paucus* [2], cropped to the extent of its ERM [23], further reveals a significant variation in habitat suitability and likely population density within the ERM polygon. The single extralimital location for *I. paucus* in (*b*) contained multiple observations.

Figure 2 expands this analysis and summarizes the results for all 59 species in our original study [2]. Figure 2*a* shows that 42.2% of the 805 235 observations occurred outside the ERMs, suggesting that ERMs are underestimating the shark distributions. Next, we crop all 50 SDM outputs to their corresponding ERM and accumulate the total species richness from all cropped SDMs (figure 2*b*). Figure 2*c* summarizes this richness within each sovereign nation's EEZ (including territories), from the global map (figure 2*b*) detailing the coastal concentration of shark species [4,7]. Figure 2*c* shows that even if we restrict our SDM outputs to the ERM extents, and do not proportionally rate SDMs by the number of market fins, we still identify the USA, Mexico, Brazil and Australia in the top 10 for shark species richness. Perhaps we agree (e.g. [4]) that the coastal ecosystems of these nations should be prioritized for global shark conservation?

**Figure 2.**
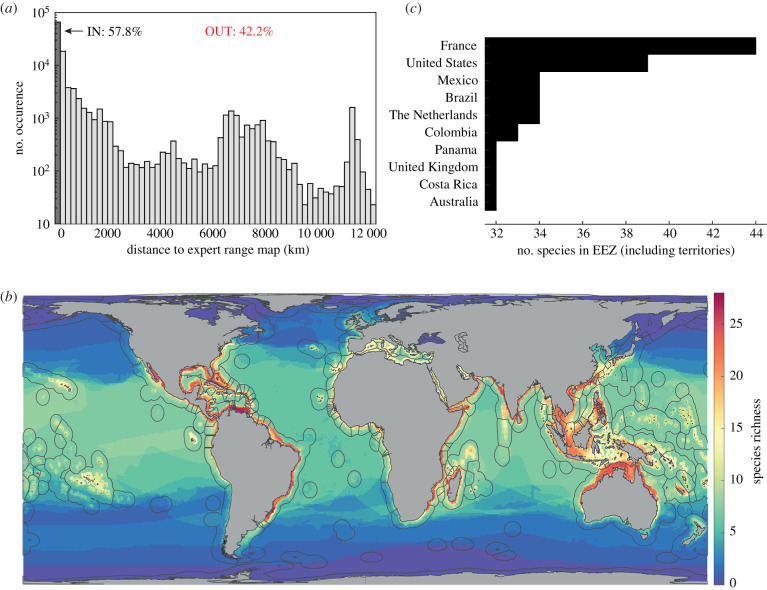
Nearly half of validated shark observations occur outside published ERMs. Aggregate results for all 59 shark species from [2] show (*a*) 42.2% of validated observations occur outside ERMs, revealing a significant gap between published range maps and occurrence data. Summarized species richness patterns from accumulating the ERM-cropped SDMs show (*b*) a coastal concentration of sharks and (*c*) the coastal zones of the USA, Mexico, Brazil and Australia as top 10 nations in shark richness.

Certainly, there remains a serious threat to oceanic sharks in today's shark fin markets, and the focus in the international community on threatened and endangered species remains critical. Our results using market identifications and SDMs add coastal species around the world as an additional important conservation focus. While the expansive distribution of many shark species does not allow us to pinpoint the exact locations of their catch, derived SDMs help to narrow the environmental niche and spatial locations where fisheries might expect the greatest interactions [21,22]. Therefore, our model conservatively assigns species' catch level to the entire area in which it most probably exists. While scalable, our approach does have limitations and can be further improved with additional data layers on marine protected areas and fishery accessibility [26], operator incentives [27] and vessel tracking [22] and market surveys. Such additions may alter our conclusions, perhaps especially by appreciating the spatial non-uniformity of fisheries catch and the importance of market proximity [26]. Until then, our analysis may be sufficient to show the large fraction of coastal sharks in the fin trade, and broadly prioritize conservation in these coastal areas while we move towards more comprehensive effort. Part of that effort should also be devoted to data acquisition and quality assurance of open access databases of species occurrence, given their utility.

Darwin himself was one of the first naturalists to appreciate that species are not fixed but change through time. While Darwin was primarily considering forms, we now know from fisheries catch, community science and tracking studies that species distributions are changing. Ocean warming is rapidly shifting ranges, as populations follow their niche across our dynamic seas. This underscores that we need contemporary data and modelling approaches to respond to this key management challenge for global shark conservation.

## Data accessibility

Published previously and available as: Van Houtan KS, Gagné TO, Reygondeau G *et al*. 2020 Data and code from: Coastal sharks supply the global shark fin trade. Open Science Framework. (https://doi.org/10.17605/OSF.IO/XVRMK) [28].
